# The Relationship Between Duration of Dialysis and Carpal Tunnel Release Outcomes: A Systematic Review

**DOI:** 10.1016/j.jhsg.2026.101054

**Published:** 2026-05-26

**Authors:** Kasra Rahmati, Pavithra Sundaravaradan, Mathangi Sridharan, Kodi Azari, Prosper Benhaim, Nirbhay S. Jain, Lauren E. Wessel

**Affiliations:** ∗ David Geffen School of Medicine, University of California Los Angeles, Los Angeles, CA; † Department of Orthopedics, University of California Los Angeles, Los Angeles, CA; ‡ Division of Plastic Surgery, Department of Surgery, University of California Los Angeles, Los Angeles, CA; § Division of Hand Surgery, Department of Plastic Surgery, University of Pittsburgh Medical Center, Pittsburgh, PA

**Keywords:** Carpal tunnel release, Carpal tunnel syndrome, Hemodialysis

## Abstract

**Purpose:**

Carpal tunnel syndrome (CTS) is a common compressive neuropathy that can become more severe in patients undergoing long-term hemodialysis (HD). Although carpal tunnel release (CTR) is the standard surgical treatment for CTS, outcomes may worsen among patients undergoing long-term HD. The aim of this systematic review is to evaluate the relationship between CTR and HD.

**Methods:**

A systematic review was conducted by screening 569 studies. Variables included duration of HD prior to CTS diagnosis, duration of symptomatic CTS, and total duration of HD therapy prior to surgical intervention. Postoperative symptom relief and complications were also collected. Studies including patients receiving peritoneal dialysis, preexisting non-HD associated CTS, or case series with n < 10 were excluded.

**Results:**

Four studies were included, consisting of 199 HD patients (271 hands). Minimum follow-up was 1 year. At 1 year, post-CTR complications were seen in 31 hands (11.4%), with the most common being persistent CTS in 25 hands (9.2%). Individuals with >10 years on HD prior to CTS diagnosis had significantly less symptomatic relief and were associated with higher rates of persistent CTS and revision when compared with those with <10 years on HD.

**Conclusions:**

CTR generally improves symptoms in HD patients with CTS. However, we demonstrated a significant inverse relationship between HD duration and symptom improvement, with higher persistence of symptoms in the long-term HD cohort. These findings underscore the importance of timely diagnosis tailored to HD duration to optimize clinical outcomes.

**Type of study/level of evidence:**

Prognostic III

Carpal tunnel syndrome (CTS) is the most common compression neuropathy, affecting approximately 3% to 6% of the general adult population and approximately 8% of the adult working population in the United States.^1^ It is often treated surgically with a carpal tunnel release (CTR), which has been increasing in incidence in the United States.^1^

For patients on hemodialysis (HD), the prevalence of CTS is higher, ranging from 25% to 40%. These rates worsen as the length of time on HD increases, as long-term HD patients are nearly 14 times more likely to develop CTS and nearly 5 times more likely to undergo CTR.^2^ As there are over 500,000 people on HD in the US, with rates increasing, this correlation will continue to impact hand surgeons in the future.^3^

Although the reasons and mechanisms for the increasing prevalence of CTS among HD patients are poorly understood, multiple hypotheses exist. There may be a correlation between the incidence of CTS in association with the laterality of the arteriovenous fistula (AVF).^4^, ^5^, ^6^ Higher AVF flow rates may lead to steal syndrome in affected arms, which may increase CTS incidence due to ischemic changes in the median nerve.^7^ As steal syndrome may occur with long-term HD treatment, this may be the underlying mechanism. However, multiple studies have reported no significant association between type of HD therapy (eg, peritoneal, AVF, central venous catheter) and CTS incidence,^4^^,^^6^ suggesting that another mechanism may be contributory, such as amyloid deposition or perturbation of nerve metabolism otherwise.^8^

In either case, although CTR may decompress the nerve, it does not reverse the underlying pathology. As such, the role of nerve decompression surgery in this setting has been debated, with proponents advocating that early decompression may slow nerve damage. Thus, we performed a systematic review to evaluate the postoperative outcomes of CTR in HD patients, focusing specifically on postoperative symptomatic relief, functional improvement, complication rates, and symptom recurrence, especially regarding timing of release in relation to initiation of dialysis.

## Methods

### Search methodology

A systematic search was conducted in PubMed, Embase, and the Cochrane Library using combinations of keywords. The search terms were used from database inception to January 30, 2025. This review was conducted in accordance with the Cochrane Handbook for Systematic Reviews of Interventions and the Preferred Reporting Items for Systematic Review and Meta-Analysis statement. Comprehensive search terms were used (Figure S1, available online on the Journal’s website at https://www.jhsgo.org).

For the purposes of this review, hemodialysis was defined as long-term or maintenance therapy, referring to chronic renal replacement therapy administered on a recurring basis for end-stage renal disease. Studies including patients receiving short-term or acute hemodialysis, defined as temporary renal replacement therapy administered for reversible acute kidney injury or as a bridge to renal transplantation, were excluded. Inclusion criteria required adult patients on maintenance HD with clinically significant CTS linked to dialysis-related pathology, details on surgical management, postoperative outcomes and revision operations, duration on hemodialysis, and a minimal follow-up period of approximately 1 year with outcomes collected at final follow-up. Studies with fewer than 10 HD subjects, without primary data, not in English, or introducing a novel surgical approach (eg, extended incision, endoscopic approach) were excluded.

Duration of hemodialysis therapy was used to stratify cohorts for subgroup analysis, with a 10-year treatment history serving as the threshold. Early cohorts were defined as <10 years (short term) on HD prior to CTS diagnosis with late cohorts defined as >10 years (long term) on HD. This cutoff was selected based on prior literature demonstrating a marked increase in morbidity, complications, and mortality beyond 10 years of hemodialysis therapy.^9^

### Data extraction and statistical analysis

Both title and abstract screening and full-text review were performed independently by 2 reviewers. Data extraction was likewise performed independently in duplicate. Discrepancies at all stages were resolved by discussion. Extracted data included HD duration prior to symptoms, CTS diagnosis, and surgery along with applicable outcomes and complications. Persistent CTS was used as a catchall for complications listed in the source articles comprising symptoms characteristic of CTS that existed at final follow-up. Source articles did not distinguish between recurrent symptoms or a failure to improve. A mixed approach of using patient-specific data where available and study-level averages otherwise was necessitated by heterogeneous reporting across patients in the included studies.

Two independent reviewers extracted data and assessed the risk of bias using the ROBINS-I tool.^10^ Meta-analysis was performed in R. Single-arm proportions were pooled under a random-effects model with the restricted maximum likelihood estimator for symptom improvement, revision rate, and overall complication rate. Heterogeneity was assessed using the *I*^2^ statistic and Cochran’s *Q* test. Subgroup analysis compared outcomes between patients with hemodialysis duration less than 10 years and greater than 10 years prior to diagnosis. Meta-regression was performed to evaluate the relationship between continuous hemodialysis duration and symptom improvement. A *P* value of <.05 was considered statistically significant.

## Results

**Figure 1:**
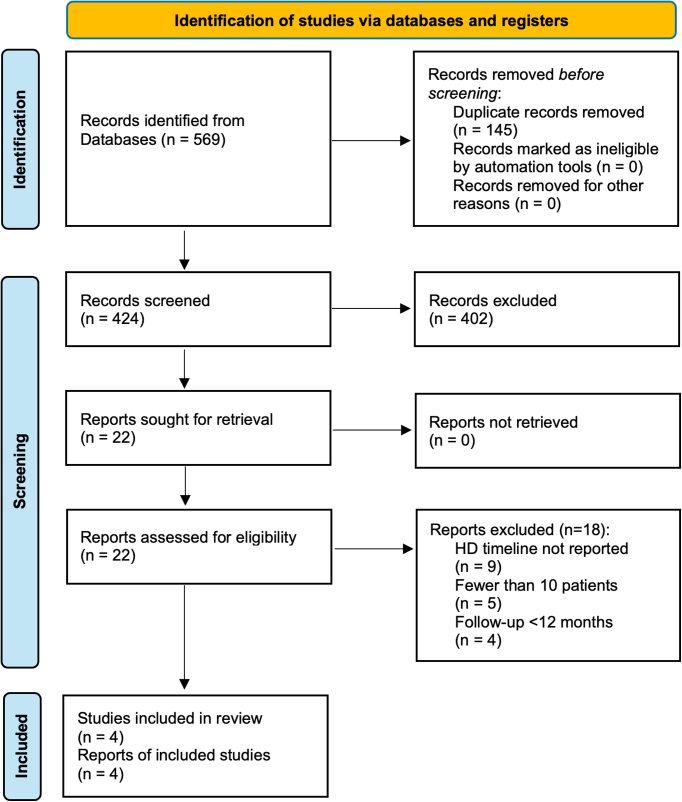
Preferred Reporting Items for Systematic Review and Meta-Analysis flow chart.

The search found 569 publications, of which 22 met the inclusion criteria for full-text review. Four studies^11^, ^12^, ^13^, ^14^ met the remaining criteria and were included (Fig. 1). These studies produced 199 HD patients (271 hands), of which 80 (40.2%) were men and 119 were women (59.8%). The mean age was 57.3 years. Rates of diabetes mellitus and body mass index were not reported.

The average time on hemodialysis, reported in 3 studies,^11^, ^12^, ^13^ before developing CTS was 10.6 years (range, 8.2–14) with an average symptomatic duration of 1.3 years (1–1.5). The average postoperative follow-up was 26.9 months (11.7–44.3) (Table 1). Symptom improvement after surgery was reported in 246 hands (90.8%), with 11.4% having complications and 9.2% having persistent symptoms (Table 2).

**Table 1:** Study Characteristics

Outcome	Kang et al^13^	Shiota et al^14^	Naito et al^11^	Gilbert et al^12^
Study design	Retrospective cohort	Retrospective cohort	Prospective cohort	Case series
Patients	36	91	31	41
Hands	44	113	43	71
Surgical technique	Open	Open	Open	Open
HD duration prior to developing CTS	14	14.8∗	9.7	8.2
Follow-up (mo)	44.3	20.4	11.7	31.0
Outcome assessment method	BCTQ administered by an independent assessor	Hamada subjective evaluation system	3-tier subjective classification	Narrative clinician assessment

BCTQ, Boston Carpal Tunnel Questionnaire; CTS, carpal tunnel syndrome; HD, hemodialysis.

∗ HD duration at time of surgery reported.

**Table 2:** Patient Demographics and CTR Outcomes in HD Patients

Outcome	Kang et al^13^	Shiota et al^14^	Naito et al^11^	Gilbert et al^12^
Age at time of surgery (y)	59.8	56.5	54.9	58.0∗
F	28 (77.8%)	48 (52.8%)	16 (52%)∗∗∗	27 (65.9%)
Sex (M:F)	1:3.5	1:1.2	1:1.1∗∗∗	1:1.9
HD duration prior to developing CTS/surgery/ and total duration of CTS (y)	14/15.5/1.5	-/14.8/-	9.7/10.7/1.0∗∗	8.2/-/-
Symptom improvement in the hands	83.3%	88.5%	95.3%	95.8%
Complications (hands)	4 (9.1%)	18 (15.9%)	2 (4.7%)	7 (9.9%)
Complication profile (hands)	2 DH, 2 rCTS	18 rCTS	2 rCTS	4 stiff, 3 rCTS
CTR to complications (mo)	12.0	-	-	-
Revisions (hands)	2 (5.6%)	12 (10.6%)	0	2 (2.8%)
CTR to revision (mo)	18.0	-	-	-

DH, delayed healing; rCTS, recurrent carpal tunnel syndrome.

All values are reported as mean unless otherwise specified.

∗ Age reported when symptoms began, not at the time of surgery.

∗∗ Total average CTS duration reported as 11.3 and 11.8 months.

∗∗∗ Ratio based on a cohort of 45 patients, which was used to estimate the remaining 31 patients who were followed up after surgery.

Two of the included studies reported an average duration on HD of less than 10 years (114 hands),^11^^,^^12^ with 2 others reporting greater than 10 years (157 hands),^13^^,^^14^ prior to CTS development. One patient was moved from the short-term cohort to the long-term cohort because of the provision of data regarding HD and revision timeline. Patients who developed symptoms greater than 10 years after the start of HD exhibited similar demographics (60% were women with an average age of 57.4 years) to those who developed symptoms less than 10 years after the start of HD (59% women and average age of 56.7 years) (Table 3).

**Table 3:** CTR Outcomes in Patients With >10 HD Versus <10 HD Years

Outcome	>10 HD years (n = 128)	<10 HD years (n = 71)	*P* value
	Kang et al^13^ and Shiota et al^14^	Naito et al^11^ and Gilbert et al^12^	
Hands	158	113	
Age at the time of surgery (y)	57.4	56.7∗	
F	77 (60.1%)	42 (59.2%)∗∗∗	
Sex (M:F)	1:1.7	1:1.7∗∗∗	
HD duration prior to developing CTS/surgery/ and total duration of CTS (y)	14/15.0/1.5	8.9/10.7/1.0∗∗	
Follow-up (mo)	27.2	23.7	
Symptom improvement in the hands	137 (87.0%)#	109 (95.6%)#	.02
Complications (hands)	23 (14.6%)	8 (7.1%)	.08
rCTS (hands)	21 (13.3%)	4 (3.5%)	<.01
Revisions (hands)	11 (7.0%)	1 (0.9%)	<.02

DH, delayed healing; rCTS, recurrent carpal tunnel syndrome.

All values are reported as mean unless otherwise specified.

# Number of hands approximated based on study-specific reported %.

∗ Age partially reported when symptoms began, not at the time of surgery.

∗∗ Total average CTS duration reported as 11.3 and 11.8 months.

∗∗∗ Ratio based on subcohort of 45 patients, which was used to estimate the remaining 31 patients who were followed up after surgery.

**Figure 2:**
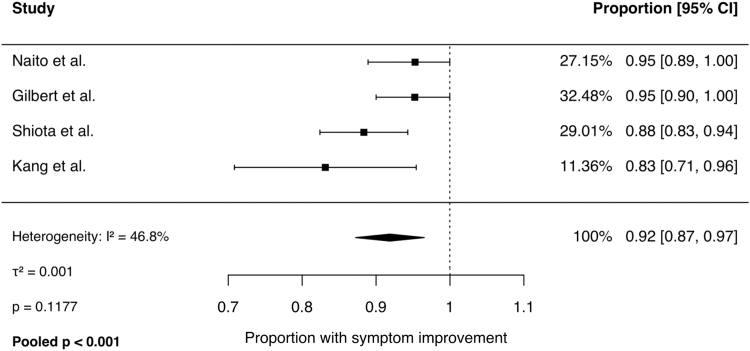
Comparison of hands with symptom improvement following CTR.

**Figure 3:**
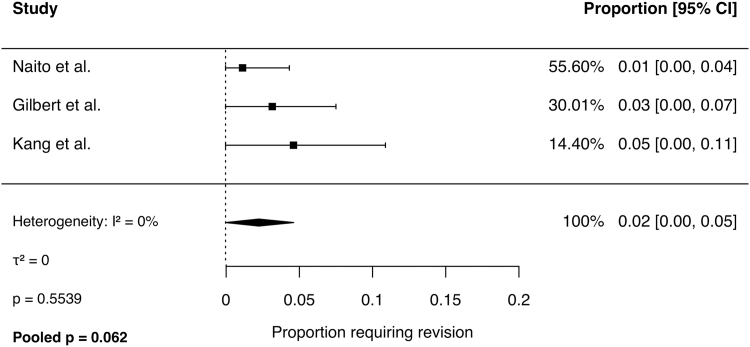
Proportion of hands requiring revision CTR.

**Figure 4:**
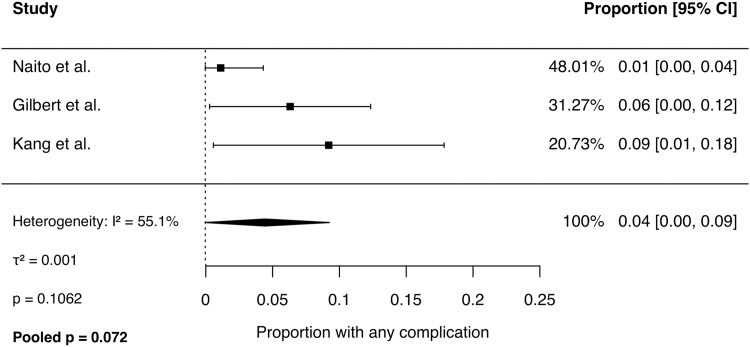
Proportion of hands with any complication following CTR.

**Figure 5:**
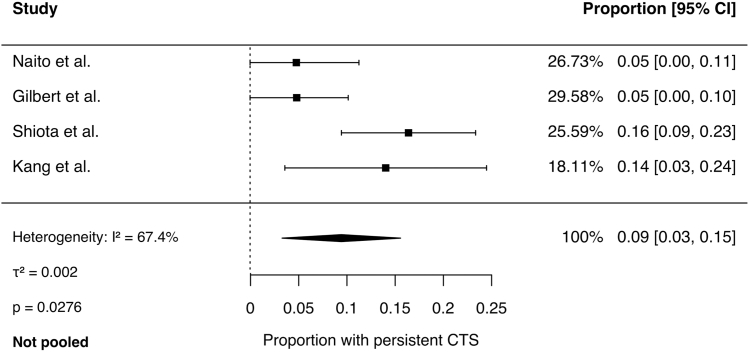
Proportion of hands with persistent CTS following CTR.

Meta-analysis of binary outcomes was performed using a random-effects model. Across 4 studies, the pooled rate of symptom improvement following CTR was 92% (95% CI, 87% to 97%; *I*^2^ = 47%, *P* = .1). The pooled revision rate across 3 studies was 2% (95% CI, 0% to 5%; *I*^2^ = 0%, *P* = .6). The pooled complication rate was 4% (95% CI, 0% to 9%; *I*^2^ = 55%, *P* = .1). Persistent CTS rates ranged from 3% to 15% across studies and was not pooled because of significant heterogeneity (*I*^2^ = 67%, *P* = .03). Forest plots for each outcome are presented in Figure 2, Figure 3, Figure 4, Figure 5.

**Figure 6:**
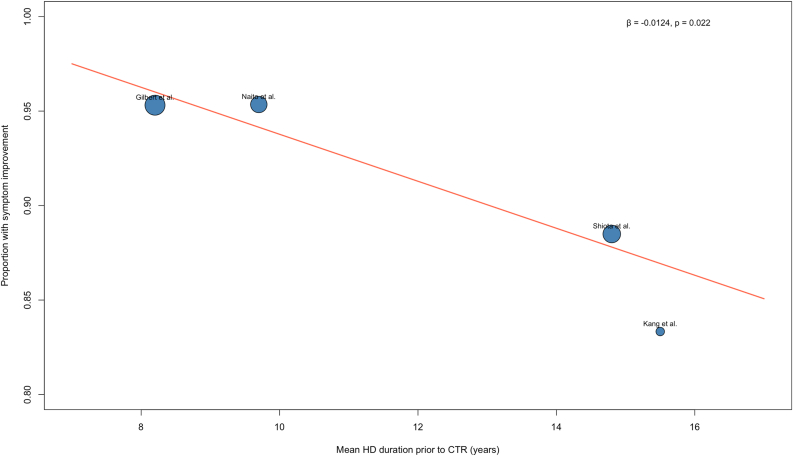
Meta-regression of HD duration and proportion with symptom improvement following index CTR.

Subgroup analysis by HD duration demonstrated that patients with less than 10 years of HD had significantly higher rates of symptom improvement compared with those with more than 10 years of HD (*P* < .01). Meta-regression displayed a significant inverse association between HD duration and symptom improvement (*β* = −0.01, *P* = .02) (Fig. 6).

Complication rates, persistent CTS, and revision rates trended lower in the short-term HD cohort, although these differences did not reach statistical significance. Of the 12 total revisions reported, heterogeneous data were provided by each study. Kang et al^13^ reported 2 revisions at 15 and 21 months following index CTR due to persistent CTS symptoms. Both patients had symptomatic improvement at 8 and 13 months following revision. Gilbert et al^12^ reported 2 cases, one due to calcified synovial-cell hyperplasia and the other due to extensive fibrosis around the median nerve with both patients reporting improved symptoms after 2- and ½-year follow-up.

## Discussion

Hemodialysis is a known risk factor for the development of CTS and may impact disease progression and treatment with CTR, although the exact relationship is unclear. In this review, we demonstrate that patients who undergo CTR in a delayed fashion after long-term HD (>10 years) experience less symptom relief compared with patients on short-term HD (<10 years). Higher rates of complications, persistent CTS, and revisions were reported within the long-term HD cohort, although not significantly more so than the short-term cohort.

Length of HD therapy was associated with increased complication rate, with 1 study reporting that patients with greater than 2 years of CTS symptoms at the time of surgery were 20% more likely to experience complications.^11^ One study also discussed patient-reported outcomes via the Boston Carpal Tunnel Questionnaire - Symptom (BCTQ-S) and Function (BCTQ-F) scores.^13^ This study presented no change in function score at 2 years after CTR, but an improvement in symptom scores, although these were well below of those who were not on HD.

Although this may reflect pathological changes to the median nerve beyond simple compressive neuropathy, the consistently high rates of symptom improvement across all studies suggest that CTR remains a highly effective intervention in HD patients and should not be regarded as futile. CTS, in the setting of HD, is theorized to be impacted by an accumulation of *β*_2_-microglobulin amyloid that, along with providing external compression on the nerve, results in irreversible changes in the nerve and progressive symptoms untreated by decompression.^8^^,^^15^, ^16^, ^17^, ^18^

There may also be a component of relative ischemia from fistula use that is alleviated with decompression, although the ischemic changes are not reversed by surgery. This may also explain the worse nerve recovery in those who were on dialysis for a longer period of time, both with and without symptoms. Laterality in relation to hemodialysis vascular access history was analyzed by one of the studies with mixed results.^12^ In select subgroups, CTS tended to align with the access-exposed limb; however, the authors concluded that vascular fistula history demonstrated varying degrees of correlation, and no conclusions could be drawn, complicating the picture of direct ischemic changes.

From a clinical standpoint, these results advocate for a proactive approach in the management of CTS among HD patients. Meta-regression analysis demonstrated a significant inverse relationship between HD duration and symptom improvement post-CTR. Thus, a high index of suspicion for CTS symptoms is reasonable, especially for those with a shorter duration of dialysis, as timely surgical intervention may yield more favorable outcomes and reduce the likelihood of persistent symptomatology and potentially lower rates of complications and subsequent revision. Both early diagnosis and intervention have a clinically significant impact on outcomes after CTR in this patient population. It is important to note that although our study suggests an association between long-term HD therapy and higher revision rates, limited revision outcomes data prevent conclusions from being drawn regarding whether these revisions were due to incomplete release, irreversible nerve damage, recurrent CTS, or other complications.

Several limitations must be acknowledged. First, only 4 studies met the inclusion criteria for detailed postoperative data, which limits the generalizability of our findings. The heterogeneity in follow-up durations (ranging from 11.7 to 44.3 months) and the variability in reporting HD timelines across studies further complicate direct comparisons. The included studies predominantly reported outcomes by hand rather than by patient, which, as the unit of analysis, may underestimate standard errors and inflate precision in patients with bilateral involvement. Furthermore, source articles did not uniformly distinguish between failure to improve and true symptom recurrence after an initial period of relief.

Outcome assessment also increased heterogeneity as Naito et al^11^ used a 3-tier subjective classification (good/fair/poor) based on patient report and clinical examination, Gilbert et al^12^ used unstructured clinician narrative assessment, Shiota et al^14^ used the Hamada evaluating system based on subjective symptom and functional status ratings, and Kang et al^13^ used the validated Boston Carpal Tunnel Questionnaire administered by an independent assessor before surgery and at each follow-up.

Advances in HD therapy such as the adoption of high-flux HD, improved filtration, and monitoring may have altered the natural history of HD-associated CTS because the included studies were conducted. Recent literature has suggested that improvement of *β*2m clearance via advances in dialysis technology might result in a significant extension in the time between starting HD and the first surgery for CTS.^19^

We note that these factors limit the direct applicability of these findings to current practice. Furthermore, we did not contact authors to retrieve missing data, given that all included studies were published between 1987 and 2012 and successful contact and data recovery were considered unlikely.

**Figure 7:**
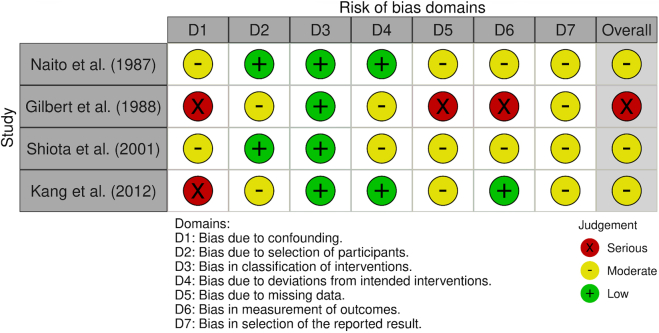
Risk of bias assessment using the ROBINS-I tool. ROBINS-I, Risk of Bias in Nonrandomized Studies of Interventions Tool.

The retrospective nature of some included studies may introduce selection bias, and the relatively small sample sizes restrict the robustness of subgroup analyses. Bias was assessed using the ROBINS-I tool; Gilbert et al^12^ were rated serious overall, whereas Naito et al,^11^ Kang et al,^13^ and Shiota et al^14^ were moderate, with confounding and outcome measurement representing the most consistent limitations across all studies (Fig. 7).

Absence of a preregistered protocol means analytic decisions were made post hoc and may be subject to reporting bias. Future research should aim to examine larger cohorts of patients on HD with standardized outcome measures and detailed reporting of HD duration relative to CTS onset and surgical outcomes. Further prospective studies would be useful in evaluating methods of early diagnosis and intervention for CTS in patients with HD.

This review demonstrates that patients with longer durations of HD prior to CTS diagnosis and CTR have lower rates of symptom relief and may have higher rates of complications and subsequent need for revisions compared with those with shorter durations on HD therapy. Patients on HD for less than 10 years have significantly higher rates of symptomatic improvement. These findings suggest that integrating HD treatment history into clinical decision making to timely screen, diagnose, and manage CTS may benefit patients receiving HD therapy. By tailoring the timing of CTS screening and potential surgical intervention to the patient’s HD history, clinicians may optimize symptomatic outcomes and potentially reduce the burden of postoperative complications in this vulnerable patient population.

## Conflicts of Interest

No benefits in any form have been received or will be received related directly to this article.
