# Supplementary figures and images for: The Relationship Between Duration of Dialysis and Carpal Tunnel Release Outcomes: A Systematic Review
Source: J Hand Surg Glob Online. 2026 May 26;8(4):101054. doi: 10.1016/j.jhsg.2026.101054 (PMC13233739; doi:10.1016/j.jhsg.2026.101054)

Figure S1. Search terms.

**
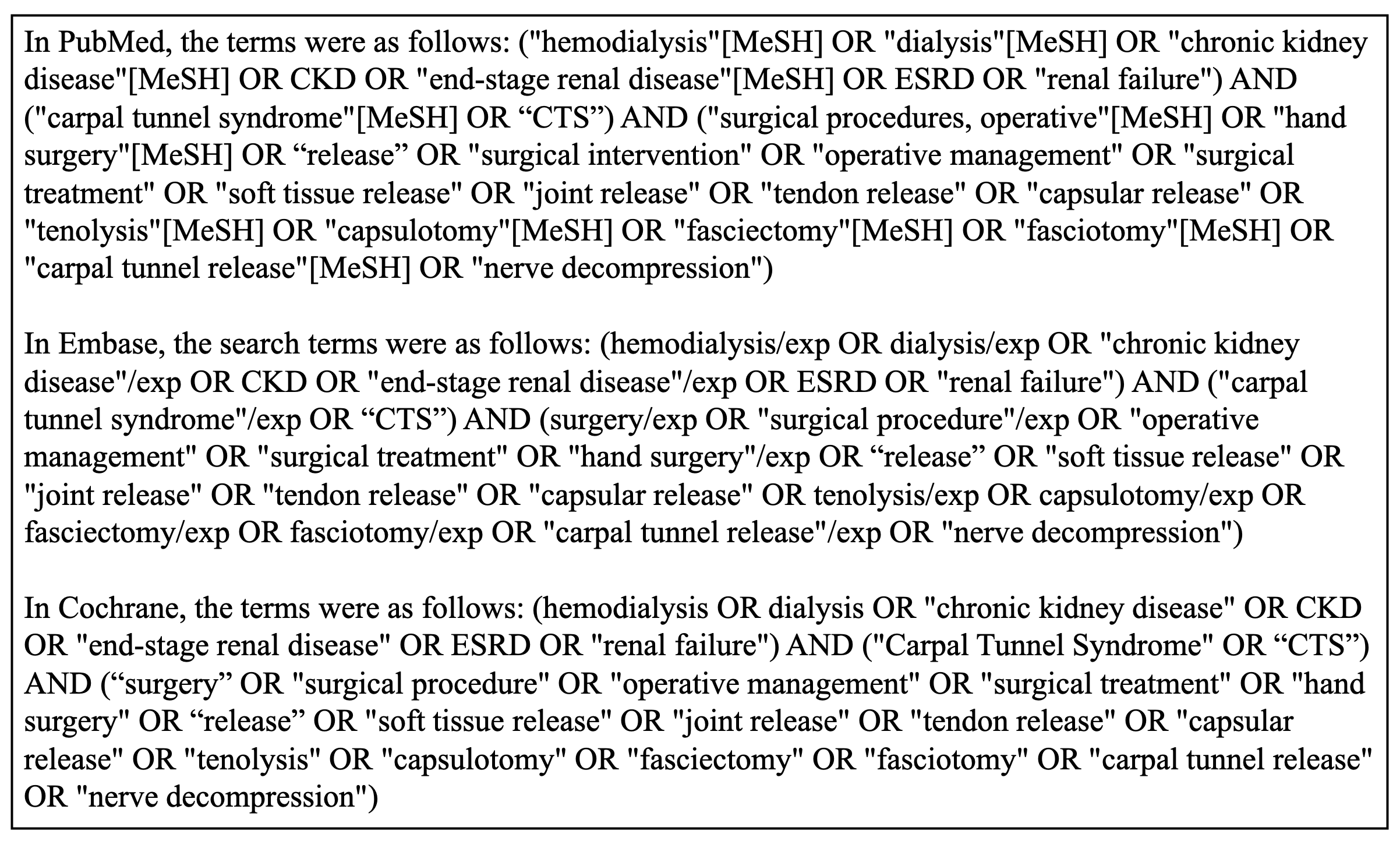
**

Supplement: Figure S1 [file mmc1.docx]
